# A multicenter study: how do medical students perceive clinical learning climate?

**DOI:** 10.3402/meo.v21.30846

**Published:** 2016-09-16

**Authors:** Nilufer Demiral Yilmaz, Serpil Velipasaoglu, Sema Ozan, Bilge Uzun Basusta, Ozlem Midik, Sumer Mamakli, Nazan Karaoglu, Funda Tengiz, Halil İbrahim Durak, Hatice Sahin

**Affiliations:** 1 Department of Medical Education, Faculty of Medicine, Ege University, Izmir, Turkey; 2 Department of Medical Education, Faculty of Medicine, Dokuz Eylül University, Izmir, Turkey; 3 Department of Medical Education, Faculty of Medicine, Hacettepe University, Ankara, Turkey; 4 Department of Medical Education, Faculty of Medicine, Ondokuz Mayıs University, Samsun, Turkey; 5 Department of Medical Education, Faculty of Medicine, Akdeniz University, Antalya, Turkey; 6 Department of Medical Education, Faculty of Medicine, Necmettin Erbakan University, Konya, Turkey; 7 Izmir University of Economics School of Vocational, Izmir, Turkey

**Keywords:** learning climate, clinical learning climate, medical students, multi-center study

## Abstract

**Background:**

The relationship between students and instructors is of crucial importance for the development of a positive learning climate. Learning climate is a multifaceted concept, and its measurement is a complicated process. The aim of this cross-sectional study was to determine medical students’ perceptions about the clinical learning climate and to investigate differences in their perceptions in terms of various variables.

**Methods:**

Medical students studying at six medical schools in Turkey were recruited for the study. All students who completed clinical rotations, which lasted for 3 or more weeks, were included in the study (*n*=3,097). Data were collected using the Clinical Learning Climate Scale (CLCS). The CLCS (36 items) includes three subscales: clinical environment, emotion, and motivation. Each item is scored using a 5-point Likert scale (1: strongly disagree to 5: strongly agree).

**Results:**

The response rate for the trainees was 69.67% (*n*=1,519), and for the interns it was 51.47% (*n*=917). The mean total CLCS score was 117.20±17.19. The rotation during which the clinical learning climate was perceived most favorably was the Physical Therapy and Rehabilitation rotation (mean score: 137.77). The most negatively perceived rotation was the General Internal Medicine rotation (mean score: 104.31). There were significant differences between mean total scores in terms of trainee/intern characteristics, internal medicine/surgical medicine rotations, and perception of success.

**Conclusion:**

The results of this study drew attention to certain aspects of the clinical learning climate in medical schools. Clinical teacher/instructor/supervisor, clinical training programs, students’ interactions in clinical settings, self-realization, mood, students’ intrinsic motivation, and institutional commitment are important components of the clinical learning climate. For this reason, the aforementioned components should be taken into consideration in studies aiming to improve clinical learning climate.

Medical education includes preclinical and clinical phases. Students feel like a physician in the clinical phase because they receive supervised clinical training from clinical instructors, interact with real patients, and encounter patients’ problems. The strengths of clinical training include being based on real-life problems and, thus, leading to motivation among learners; and learners being able to observe trainers’ professional thoughts, behaviors, and attitudes during training (1, 2). In several studies, students stated that their expectations pertaining to clinical training were as follows: increased responsibility, regular observation of their work by trainers, opportunities to implement technical and problem-solving skills, to quickly and easily receive responses to problems, to give feedback, to receive support and motivation, to be part of a team, mutual positive relationships and respect, and harmony in the team (3–7). However, clinical training is rather distressing due to the following factors: patient care, conflicts between service delivery and training requirements, large groups of students, improper learning conditions of hospitals, inequality of opportunity, low motivation and poor training skills of teachers, inadequate feedback, lack of resources, negative attitudes displayed by the staff toward students, and negative learning climate (2, 8–10). Undergraduate medical education is marked by numerous transitions, which range from arrival at the medical school to frequent changes in clinical settings or the adoption of clinical responsibility for patients. Transitions are often marked by increased anxiety due to the disruption of usual routines and social contacts, as well as a perceived threat to the present situation (11).

Learning climate refers to the quality of the communication between students and instructors and involves students’ active participation in the learning process, students’ academic expectations, and a safe and respectful atmosphere for everyone in the school (12). When evaluating the learning climate of medical schools, the preclinic phase and the clinic phase should be examined separately (13). In this article, the concept of learning climate refers to the clinical learning climate.

Effective clinical training is focused on two key elements: patient care and the establishment of a positive clinical learning climate (14). The clinical learning climate is defined as an interactive network of forces in the clinical setting that influences students’ clinical learning (15). Interactions between students and instructors are of paramount importance for the establishment of a positive learning climate. According to Dunn and Hansford, students’ satisfaction is greatly affected by a positive learning climate (16). The learning climate plays a critical role in successful education by supporting the process of learning (17, 18).

Learning climate is a multifaceted concept that is complicated to measure. Numerous scales have been developed for the assessment of learning climates in medical education (19). The scales that are currently available are used to assess either the overall learning climate (20–24) or the clinical phase alone. The scales that are used to assess only the clinical phase are the following: Manchester Clinical Placement Index-MCPI; Clinical Learning Climate Scale-CLCS; the Undergraduate Clinical Phase Environment Measure-UCEEM; Clinical Learning Climate Scale-CLCS (2010), which was developed by Demiral YIlmaz; and the Postgraduate Hospital Educational Environment Measure (PHEEM) (25–29).

In this study, it was aimed to determine the perceptions of students about the clinical learning climate in terms of gender, age, the type of education program, status of attending the traineeship or internship period, group of rotations, and perception of success.

## Methods

This is a cross-sectional study which recruited students from six well-established medical schools in four different geographical regions of Turkey. In Turkey, medical schools including premedical education takes 6 years (12 semesters). The first 3 years (1st–6th semesters) comprise the preclinical phase, whereas the latter 3 years (7th–12th semesters) comprise the clinical phase. The first four semesters of clinical training are called the traineeship period, and students in this period are called trainees. The last two semesters are called the internship period, and students in this period are called interns. During the traineeship period, students develop their medical knowledge and skills through clinical rotations. They also observe the diagnosis/treatment process under the supervision of faculty members. During the internship period, work-based training is conducted and students develop their medical knowledge and skills and professional values under the supervision of faculty members. Education programs are structured in a way so as to allow horizontal and vertical integration of preclinical and clinical phases. While two of the medical schools participating in the study implemented task-based learning (TBL) during the clinical period, the remaining four medical schools implemented the system-based integrated program.

This study was conducted with clinical-phase students attending medical schools at A, B, C, D, E, and F universities. All students who completed clinical rotations, which lasted for 3 or more weeks, were included in the study (*n*=3,097). The number of trainees and interns recruited for the study was 2,180 and 917, respectively.

Cardiology, Dermatology, Infectious Diseases, General Internal Medicine (including hematology/oncology, nephrology, gastroenterology, endocrinology, diabetes and metabolism, rheumatology, allergy and immunology, and geriatric medicine), Neurology, Pediatrics, Physical Medicine and Rehabilitation, Psychiatry, and Pulmonary Diseases were classified as rotations of internal medicine. On the other hand, Anesthesia, Emergency Medicine, Obstetrics and Gynecology, Ophthalmology, Otology and Laryngology, Pediatric Surgery, and General Surgery were classified as rotations of surgical medicine (Fig. 2). Rotations of General Internal Medicine, General Surgery, Obstetrics and Gynecology, and Pediatrics were deemed major rotations. In the curricula of the medical schools in Turkey, major rotations are mandatory for all students in the traineeship and internship phases.

The duration of major rotations is lengthy, and the education objectives of these rotations provide a basis for remaining rotations.

### Instruments

**Fig. 1:**
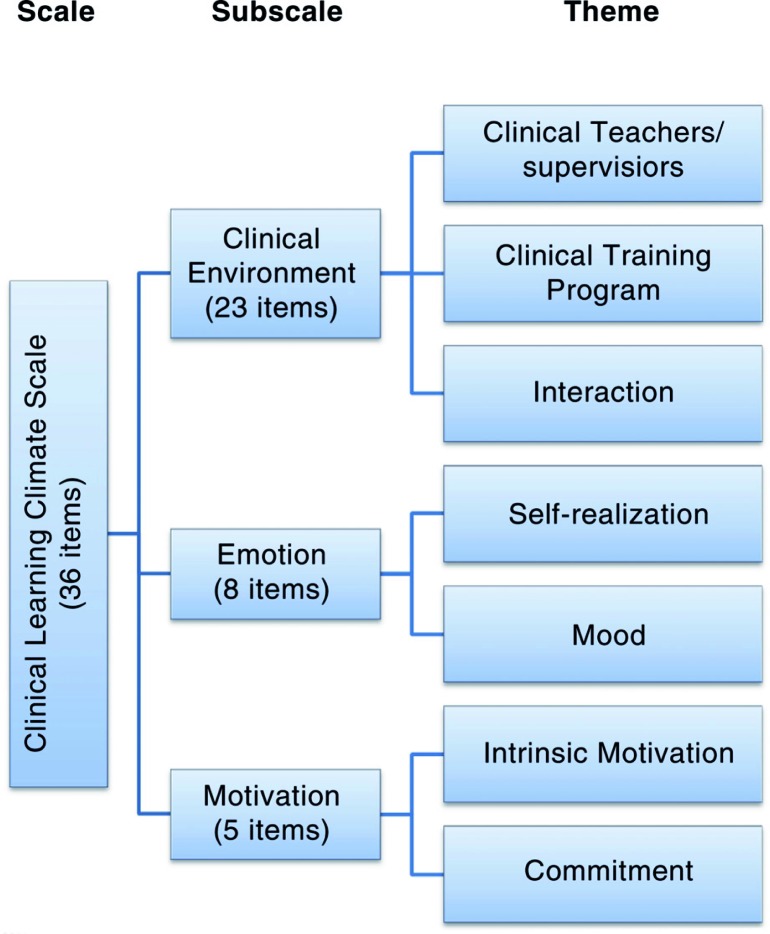
CLCS in terms of subscales and themes.

In data collection, the CLCS was used (which was developed by Demiral Yilmaz (28) with data collected from trainees and interns, similar to the present study). Demiral Yilmaz also performed the validity and reliability study of the scale (Cronbach α: 0.92) (28). The CLCS includes 36 items (see Appendix). The scale has three subscales: clinical environment (23 items), emotion (eight items), and motivation (five items). The clinical environment subscale measures the following themes: clinical teacher/instructor/supervisor (CT/S), clinical training program (CTP), and students’ interactions (IA) in clinical settings. The emotion subscale includes items on self-realization (SR) and mood (Mo). The motivation subscale includes items questioning students’ intrinsic motivation (IM) and institutional commitment (IC) (Fig. 1).

Each item in the CLCS is scored using a 5-point Likert scale (1: strongly disagree to 5: strongly agree). Three items (# 5, # 19, # 26) are reversely encoded. The lowest and highest scores that can be obtained from the scale are 36 and 180, respectively. There is no cutoff value for the scale. Higher scores suggest that the clinical learning climate was positively perceived. In addition to the CLCS, information on which medical school students study at, students’ age and gender, whether the student is attending the traineeship period or the internship period, and the name of the clinical rotation were gathered using open-ended questions. Data pertaining to the perception of success (student's perception of their own performance) were questioned under three categories (bad, moderate, and good).

The approximation rate to the maximum score (ARMS) to be obtained from the overall scale and from the subscales was calculated using the following formula: reported score/expected score×100. Higher ARMS values indicated that the perception of clinical learning climate regarding the overall scale and subscales was positive.

### Data collection and analysis

The students were verbally informed on the aim of the study and invited to participate in the study. Voluntary informed consent was obtained from the students who accepted the invitation to the study. Data were collected in a single session on the last day of rotations under the supervision of the researchers. Students from each medical school marked their responses on the CLCS that was presented to them as a hard copy document (self-report technique). Data were collected between December 2011 and March 2012.

Data collected from the medical schools were entered into a standard database by the researchers in the relevant medical school. Each medical school only used the data belonging to the students who marked each question, and scales that were incomplete were excluded from the study (data quality control). Thus, data were made to be more homogeneous. Data were gathered from these databases and analyzed at a single center. Data were adjusted by age and then were analyzed by grouping the internal medicine rotations (Cardiology, Dermatology, Infectious Diseases, General Internal Medicine, Neurology, Pediatrics, Physical Medicine and Rehabilitation, Psychiatry, and Pulmonary Diseases) and surgical medicine rotations (Anesthesia, Emergency Medicine, Obstetrics and Gynecology, Ophthalmology, Otology and Laryngology, Pediatric Surgery, and General Surgery). To analyze the data, descriptive statistics, *t*-tests, one-way ANOVA, and post-hoc tests (Scheffé test) were used. The *p*-value was accepted statistically significant at the alpha value of 0.05. Statistical analysis was performed using SPSS for Windows (SPSS, Inc. IBM) Version 18.0.

### Ethical considerations

The study was approved by the Ethics Committee of Ege University Faculty of Medicine (Decision Number: 11-6.1/10).

## Results

The response rate for the trainees was 69.67% (*n*=1,519), whereas for the interns it was 51.47% (*n*=917). The mean age of the students was 22.96±1.31 (min: 20 – max: 32). Among the students, 52.60% were male and 94.0% had a moderate or good level of perception of success.

**Fig. 2:**
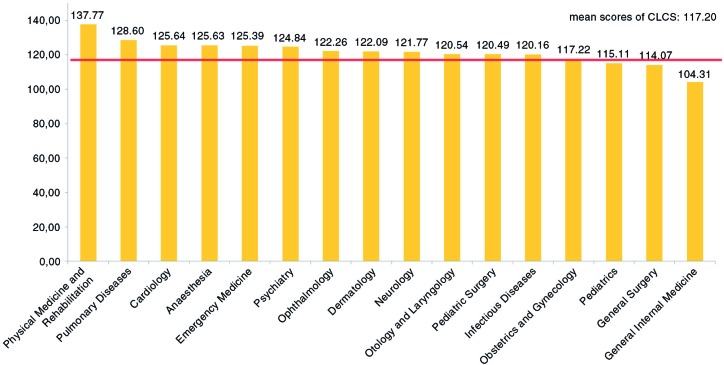
The mean CLCS scores according to rotations.

The mean total CLCS score was 117.20±17.19 (min: 39 – max: 180). The rotation in which the clinical learning climate was perceived most favorably was the Physical Therapy and Rehabilitation rotation (mean: 137.77). The most negatively perceived rotation was the Internal Medicine rotation (mean: 104.31). The mean score pertaining to major rotations (Obstetrics and Gynecology, Pediatrics, General Surgery, and General Internal Medicine) was similar to the mean total CLCS score (Fig. 2).

A significant difference was found between mean total CLCS scores pertaining to the Physical Therapy and Rehabilitation rotation and the General Internal Medicine rotation (*t*=9.25; *p*=0.000).

Table 1 presents a comparison of total scores and subscale scores in terms of gender, age, the type of education program, status of attending traineeship or internship, group of rotations, and perception of success.

**Table 1:** Mean total CLCS and subscale scores in terms of student characteristics

		CLCS		Clinical environment		Emotion		Motivation	
					
		Mean±SD	*t*/*F*^a^ (*p*)	Mean±SD	*t*/*F*^a^ (*p*)	Mean±SD	*t*/*F*^a^ (*p*)	Mean±SD	*t*/*F*^a^ (*p*)
Gender	Female	118.08±20.54	1.80 (0.07)	74.46±16.17	1.14 (0.25)	23.81±7.20	1.10 (0.27)	19.80±2.66	**3.93 (0.00)**
	Male	116.38±21.29		73.63±16.46		23.45±7.04		19.29±3.06	
Age	20–24	117.05±20.74	0.88 (0.37)	73.92±16.18	0.79 (0.42)	23.61±7.14	0.16 (0.86)	19.50±2.88	1.50 (0.13)
	25–29	118.43±22.60		74.90±17.59		23.70±6.93		19.82±2.88	
Type of education program	TBL	118.09±21.92	1.29 (0.19)	73.86±16.96	0.32 (0.74)	24.52±7.54	**3.85 (0.00)**	19.70±2.76	1.75 (0.07)
	SBIP	116.78±20.47		74.11±16.02		23.20±6.88		19.45±2.93	
Trainee/intern	Trainee	118.47±20.85	**4.90 (0.00)**	75.19±16.01	**5.75 (0.00)**	23.78±7.19	1.74 (0.08)	19.49±2.91	1.21 (0.22)
	Intern	113.08±20.75		70.28±16.77		23.12±6.87		19.67±2.79	
Rotation	Internal medicine	112.62±20.30	**2.29 (0.02)**	74.40±16.06	1.22 (0.22)	22.19±7.05	**11.08 (0.00)**	19.47±2.86	1.14 (0.25)
	Surgical medicine	114.80±19.79		73.49±16.69		25.69±6.70		19.62±2.91	
Perception of success	Poor	110.31±21.84	**12.30^a^ (0.00)**	71.59±16.84	**3.13^a^ (0.04)**	21.02±7.16	**10.79^a^ (0.00)**	17.69±3.43	**61.31^a^ (0.00)**
	Moderate	116.10±20.83		73.51±16.18		23.45±6.95		19.13±2.90	
	Good	119.24±20.70		74.90±16.37		24.14±7.21		20.20±2.59	

CLCS: Clinical Learning Climate Scale, SBIP: system-based integrated program, TBL: task-based learning.

a One-way ANOVA test.

Bold values indicate statistical significant values (*p*<0.05).

There were significant differences in the mean total CLCS scores in terms of trainee/intern characteristics, rotation, and perception of success. The mean total CLCS score was significantly higher in trainees (118.47±20.85) than in interns, in surgical medicine rotations (114.80±19.79) than in internal medicine rotations, and in students with a good level of perception of success (119.24±20.70).

There were significant differences in the mean *clinical environment* subscale scores in terms of trainee/intern characteristics (*t*=5.75; *p*=0.000) and perception of success (*F*=3.13; *p*=0.040). The trainees (75.19±16.01) and students who reported a good level of perception of success (74.90±16.37) obtained higher scores.

There were significant differences in the mean *emotion* subscale scores in terms of the type of education program (*t*=3.85; *p*=0.000), group of rotations (*t*=11.08; *p*=0.000), and perception of success (*F*=10.79; *p*=0.000). Students in the TBL program (24.52±7.54), those studying in surgical medicine rotation (25.69±6.70), and those who reported a good level of perception of success (24.14±7.22) obtained significantly higher scores.

There were significant differences in the mean *motivation* subscale scores in terms of gender (*t*=3.93; *p*=0.000) and perception of success (*F*=61.31; *p*=0.000). In the motivation subscale, female students had a higher mean score compared with male students. In addition, students with higher success perception had a higher mean score compared to other students.

**Fig. 3:**
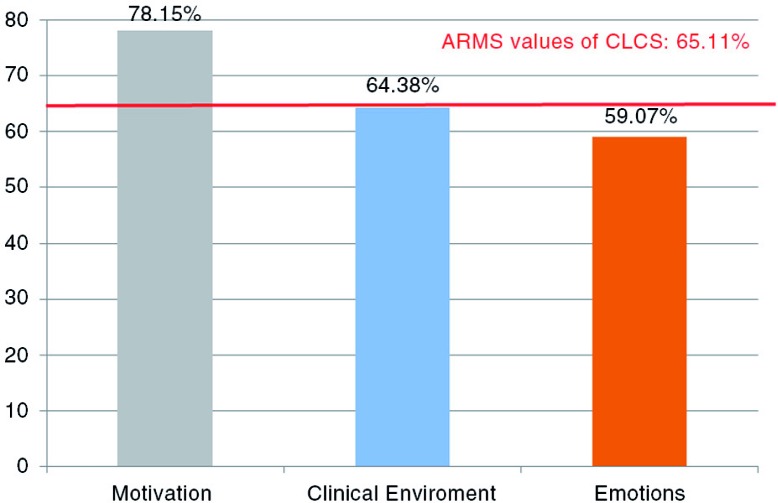
ARMS values for the subscales.

The ARMS value was 65.11% for the CLCS, whereas the value was 78.15% for the *motivation* subscale, 64.38% for the *clinical environment* subscale, and 59.07% for the *emotion* subscale (Fig. 3).

**Fig. 4:**
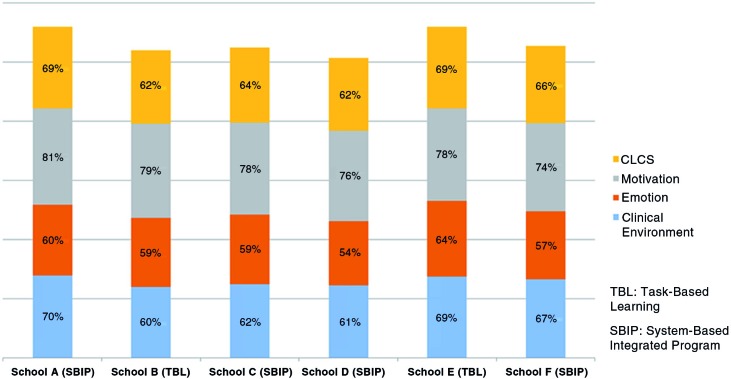
ARMS values for the medical schools.

ARMS values were calculated according to medical schools (Fig. 4). ARMS values for the CLCS in different medical schools were similar (62–69%). However, regarding the ARMS values for the subscales, the emotion subscale scores were lower in all medical schools (54–64%). The highest ARMS value was determined in the motivation subscale for all medical schools.

## Discussion

In this study, clinical learning climate perceptions of students who were attending six different medical schools in Turkey were examined.

The mean CLCS score found in this study was compared with those found in previous studies. This study revealed that gender, age, and the type of education program were not associated with students’ perception about the clinical learning climate. In Boor's study, no difference was determined between males and females in terms of learning climate (18). Similar to the results of this study, Clapham, Roff, Filho and Gough reported that female students’ mean scores for the learning climate were found to be higher compared with male students (29–32).

Students who attended medical schools where the TBL program was implemented significantly scored higher on the *emotion* subscale. In two of the medical schools where the TBL program was implemented, the number of students per faculty member in clinical sciences was 3.64 and 4.03, whereas this rate ranged from 4.68 to 5.07 in other medical schools (33). The number of students per faculty member in the medical schools where the TBL was implemented may have influenced the emotion subscale scores. It was also reported that the multidisciplinary teaching/learning setting in which the TBL encourages students to take their own responsibility and supports appropriate learning climate contributes to students’ positive perceptions (34).

In this study, it was determined that the trainees perceived the clinical learning climate more positively than the interns did. This finding is consistent with the findings of other studies indicating the view that medical students’ perceptions about learning climate change for the worse as their years at school increase (21, 35–38). There may be various factors leading to interns’ negative perceptions concerning clinical learning climate. During the internship period, unlike previous years, students are supposed not only to learn but also to take responsibility. The training program during the internship period is less structured. Interns comply with working conditions of the rotations where they receive their training and they are ‘residents’ who often feel as if they are in the lower rungs of the hierarchy (39). Because the job description changes from one clinical rotation to another and standards used for achievement evaluation are inadequate, students face uncertainty and experience high levels of anxiety during internship. In Turkey, medical school graduates have to take an examination in order to specialize in one area of medicine. The Specialization Examination in Medicine may cause interns to perceive clinical learning climate as unfavorable.

The mean CLCS scores and the mean *emotion* subscale scores in surgical medicine rotations were significantly higher. There were no significant differences in mean *clinical environment* subscale scores in terms of internal medicine rotations. It was thought-provoking that while the students had positive perceptions about rotations, they negatively perceived the clinical learning climate during surgical medicine rotations. Although it appears to be contradictory, this situation can be explained by the fact that the results of surgical interventions can be quickly seen, which enables students to develop a positive sense of professional satisfaction.

In this study, while the lowest mean total CLCS score was obtained from the General Internal Medicine rotation, the highest mean total CLCS score was obtained from the Physical Therapy and Rehabilitation rotation. The mean *clinical environment* and *emotion* subscale scores pertaining to the Physical Therapy and Rehabilitation rotation were higher compared with the Internal Medicine rotation. In Turkey, differences in patient loads of rotations, students’ patient-related responsibilities, and faculty–student interactions are thought to lead to different perceptions of emotions and clinical environments.

In the study of van Der Hem-Stookros, medical students displayed negative perceptions concerning clinical learning climate during the general surgery rotation (35).

The results of this study regarding the major rotations are different from those found in the Association of American Medical Colleges (AAMC) surveys, which are conducted with graduate students every year. According to the data provided by the AAMC in 2014, students obtained the highest mean scores for the quality of their educational experiences in clinical rotations during the Internal Medicine rotation, whereas they obtained the lowest scores during the Obstetrics and Gynecology rotation (40).

As noted by Boor, further studies are needed on factors that are associated with students’ perceptions regarding rotations because characteristics of each rotation affect the clinical learning climate (41). Comparison of the students’ perceptions regarding clinical learning climate and their success revealed that the higher their perception of success was, the more favorable was their perception of clinical learning climate. These results are consistent with the results of studies emphasizing that a positive learning climate promotes students’ success and morale (42–45). This view is also consistent with Freiberg's work which highlights the importance of learning climate: ‘climate is not only an effective element of learning but it also brings about success’ (46).

In this study, the ARMS of the students’ perceptions about the clinical learning climate was 65.11%. Various studies on learning climate revealed that the ARMS of the assessment instrument was lower than that found in this study and that the ARMS ranged between 39 and 59% (30, 32, 36, 47–50). In the study by Roff, the ARMS rate was similar (65%) (30). On the other hand, the ARMS rate was higher in other studies and ranged between 66 and 72% (30, 37, 48, 51). ARMS rates increase as students’ expectations regarding the clinical learning climate are met. Thus, unmet expectations provide information about aspects of the clinical learning climate that need improvement.

Evaluation of the students’ perceptions about the clinical learning climate in terms of the subscales revealed that the ARMS for the *motivation* subscale was higher compared to the other two subscales. Previous studies that were conducted with medical students yielded results similar to the findings of studies carried out with dental students. Henzi reported that interest in the profession was one of the most positively perceived dimensions related to the clinical learning climate (52).

In this study, the subscale with the lowest ARMS value was the *emotion* subscale. Similarly, in studies conducted by Al-Hazimi, and Ahmed, the most negatively perceived subscale related to the learning climate was the *emotion* subscale (48, 53). According to Bandura, affective responses are important because they help a learner to acquire necessary knowledge and skills through mastery, observation of others, feedback, and support (54). Low mean scores obtained from the *emotion* subscale indicate the presence of problems in this area, which suggests that medical schools should make efforts toward improvement regarding this subscale.

## Conclusion

The results of this study drew attention to certain aspects of the clinical learning climate in medical schools. The scales used for data collection were found to be appropriate. The ARMS values found in the study may provide information about areas to be studied in the future. Since this was a multicentered study which involved various medical schools with different education programs, a large sample size was reached and the evidential value of the findings increased. Although the names of rotations across medical schools were the same, differences in curricula led to differences in clinical learning climates. Another noteworthy contribution of this study was that data observed in the present study reflect the real-life situation pertaining to the clinical learning climate.

Clinical learning climate is a multifaceted concept that is complicated to measure (19). CT/S, CTP, students’ IA in clinical settings, SR, Mo, students’ IM, and IC are all important components for the clinical learning climate. For this reason, the aforementioned components should be taken into consideration in studies aiming to improve clinical learning climate.

Through the measurement of the clinical learning climate, the expectations of the students are determined, and students can participate in the decision-making process. By taking feedback from the instructors and students, information on the quality of clinical education is also obtained. The strengths and weaknesses of clinical education can be determined. Additionally, the clinical learning climate can be monitored, and institutions can be compared on local, regional, and national levels (25, 29).

In future studies, it is suggested that the studies should be multicentered, each component of the clinical learning climate should be examined, and all students in their clinical phase should be encouraged to participate, including qualitative data in the studies.

The strength of this multicenter study was that it was conducted in well-established medical schools with different types of education programs. The limitation of the study was that although the response rate was high, the number of interns participating in the study was low.

## Appendix

**Table:** The Clinical Learning Climate Scale

Item no.	Subscale	Theme	Items
1	CE	CT/S	Clinical instructors are enthusiastic while they provide training in the rotation.
2	CE	CT/S	Clinical instructors have good teaching skills.
3	M	IM	I enjoy giving care to patients.
4	E	SR	During this rotation/internship, I had enough time to study on my own.
5*	E	Mo	Students are depressed during this rotation/internship.
6	M	IM	I have good relationships with my friends at school.
7	CE	CT/S	In this rotation, clinical instructors are understanding toward the students and are attentive to their relationship with the students.
8	CE	CT/S	In this rotation, specialists/assistants are satisfied with their role and responsibility in students’ training.
9	CE	CT/S	In this rotation, clinical instructors teach the necessary information related to patient care.
10	CE	CTP	In this rotation, students’ performances are assessed fairly.
11	M	IM	I want to become a doctor.
12	E	Mo	During this rotation/internship, I felt healthy.
13	CE	CT/S	Clinical instructors behave patiently if the students in the rotation fail to know a topic.
14	CE	CT/S	Clinical instructors are open-minded toward students’ opinions and thoughts in the rotation.
15	CE	CTP	In this rotation, clinical instructors clearly indicate what is expected from the students during the learning process in advance.
16	CE	IA	In this rotation, specialists/assistants show respect to the students.
17	M	IM	I am willing to learn issues related to my profession.
18	E	SR	During this rotation/internship, I had enough time to relax.
19*	E	Mo	Students are tense during this internship.
20	CE	CT/S	In this rotation, clinical instructors are good role models in terms of ethics.
21	CE	IA	In this rotation, clinical instructors show respect to the students.
22	CE	IA	In this rotation, students can easily access specialists/assistants when they need help.
23	CE	CTP	In this rotation, students receive good education.
24	CE	CTP	In this rotation, achievement levels expected from the students are attainable.
25	E	SR	I was able to take some time to myself during this rotation/internship.
26*	E	Mo	Students are anxious during this rotation/internship.
27	CE	CT/S	In this rotation, clinical instructors believe that the students bear responsibility for learning.
28	CE	CTP	In this rotation, equipment and materials to be used in practices can be easily accessed.
29	E	Mo	During this rotation/internship, I felt exhausted.
30	M	IC	I am proud of my school.
31	CE	CT/S	In this rotation, clinical instructors believe wholeheartedly that each student will be successful.
32	CE	IA	In this rotation, students can easily access faculty members when they need help.
33	CE	IA	In this rotation, relationships between superordinates and subordinates are not strict; there is an equalitarian atmosphere.
34	CE	IA	In this rotation, all students are treated equally.
35	CE	CTP	Questions asked in the exam(s) in this rotation have been designed to measure all the learning objectives.
36	CE	CT/S	In this rotation, clinical instructors give good service to patients.

* Reversely encoded.

CE: Clinical environment, E: Emotion, M: Motivation, CT/S: clinical teacher/instructor/supervisor, CTP: clinical training program, IA: students’ interactions in clinical settings, SR: self-realization, Mo: mood, IM: students’ intrinsic motivation, IC: institutional commitment.

## References

[1] Rolfe IE, Sanson-Fisher RW (2002). Translating learning principles into practice. Med Educ.

[2] Spencer J (2003). ABC of learning and teaching in medicine: learning and teaching in the clinical environment. BMJ.

[3] Robins LS (1996). A model of student satisfaction with the medical school learning environment.

[4] Parsel G, Bligh J (2001). Recent perspectives on clinical teaching. Med Educ.

[5] Chan DS, Ip WY (2007). Perception of hospital learning environment: a survey of Hong Kong nursing students. Nurse Educ Today.

[6] Kendall ML, Hesketh EA, Macpherson SG (2005). The learning environment for junior doctor training – what hinders, what helps. Med Teach.

[7] Ramani S, Leinster S (2008). AMEE Guide no. 34: teaching in the clinical environment. Med Teach.

[8] Seabrook MA (2004). Clinical students’ initial reports of the educational climate in a single medical school. Med Educ.

[9] Dolmans DH, Wolfhagen IH, Heineman E, Scherpbier A (2008). Factors adversely affecting student learning in the clinical learning environment: a student perspective. Educ Health.

[10] AMA (2012). Junior doctor training, education and supervision survey, March 2013.

[11] Tsai JC, Chen CS, Sun IF, Liu KM, Lai CS (2014). Clinical learning environment measurement for medical trainees at transitions: relations with socio-cultural factors and mental distress. BMC Med Educ.

[12] Cohen J (2006). Social, emotional, ethical and academic education: creating a climate for learning, participation in democracy and well-being. Harv Educ Rev.

[13] Demiral Yılmaz N, Velipasaoglu S, Sahin H, Uzun Basusta B, Midik O, Coskun O (2016). A de novo tool to measure the preclinical learning climate. Educ Sci Theory Pract.

[14] Sheehan D, Wilkinson TJ, Billett S (2005). Interns’ participation and learning in clinical environments in a New Zealand hospital. Acad Med.

[15] Dunn SV, Burnett P (1995). The development of a clinical learning environment scale. J Adv Nurs.

[16] Dunn SV, Hansford B (1997). Undergraduate nursing students’ perceptions of their clinical learning environment. J Adv Nurs.

[17] Roff S, McAleer S (2001). What is educational climate?. Med Teach.

[18] Boor K, Scheele F, van der Vleuten CP, Scherpbier AJ, Teunissen PW, Sijtsma K (2007). Psychometric properties of an instrument to measure the clinical learning environment. Med Educ.

[19] Lombarts KM, Heineman MJ, Scherpbier AJJA, Arah OA (2014). Effect of the learning climate of residency programs on faculty's teaching performance as evaluated by residents. PLoS One.

[20] Marshall RE (1978). Measuring the medical school learning environment. J Med Educ.

[21] Polali L, Price J (2000). Developments: validation and use of an instrument to measure the learning environment as perceived by medical students. Teach Learn Med.

[22] Roff S, McAleer S, Harden RM, Al-Qahtani MA (1996). Development of a validated Medical Education Environment Measure (MEEM).

[23] Roff S, McAleer S, Harden RM, Al-Qahtani M, Ahmed AU, Deza H (1997). Development and validation of the Dundee Ready Education Environment Measure (DREEM). Med Teach.

[24] Roff S (2005). The Dundee Ready Educational Environment Measure (DREEM) – a generic instrument for measuring students’ perceptions of undergraduate health professions curricula. Med Teach.

[25] Dornan T, Mann K, Scherpbier A, Spencer J, Norman G (2011). Medical education theory and practice.

[26] Wangsaturak D, McAleer S (2008). Development of the clinical learning climate measure for undergraduate medical education. South East Asian J Med Educ.

[27] Strand P, Sjöborg K, Stalmeijer R, Wichmann-Hansen G, Jakobsson U, Edgren G (2013). Development and psychometric evaluation of the Undergraduate Clinical Education Environment Measure (UCEEM). Med Teach.

[28] Demiral Yılmaz N (2010). The examination of medicine students’ learning climate perceptions regarding the academical self-efficacy, attitude towards medicine occupation and academical success (Turkish).

[29] Clapham M, Wall D, Batchelor A (2007). Educational environment in intensive care medicine – use of Postgraduate Hospital Educational Environment Measure (PHEEM). Med Teach.

[30] Roff S, McAleer S, Ifere OS, Bhattacharya S (2001). A Global diagnostic tool for measuring educational environment: comparing Nigeria and Nepal. Med Teach.

[31] Filho GRO, Vieira JE, Schonhorst L (2005). Psychometric properties of the Dundee Ready Educational Environment Measure (DREEM) applied to medical residents. Med Teach.

[32] Gough J, Bullen M, Donath S (2010). PHEEM ‘downunder’. Med Teach.

[33] Undergraduate Medical Education Report 2010 – Mezuniyet Öncesi TIp Eğitimi Raporu 2010 (Turkish).

[34] Harden R, Crosby J, Davis MH, Howie PW, Struthers AD (2000). Task-based learning: the answer to integration and problem based learning in the clinical years. Med Educ.

[35] van der Hem-Stookros HH, Scherpbier A, van Der Vleuten CP, De Vries H, Haarman HJ (2001). How effective is a clerkship as a learning environment. Med Teach.

[36] Jiffry MT, McAleer S, Fernando S, Marasinghe RB (2005). Using the DREEM questionnaire to gather baseline information on an evolving medical school in Sri Lanka. Med Teach.

[37] Miles S, Leinster S (2007). Medical students’ perceptions of their educational environment: expected versus actual perceptions. Med Educ.

[38] Riquelme A, Oporto M, Oporto J, Mendez JI, Viviani P, Salech F (2009). Measuring students’ perceptions of the educational climate of the new curriculum at the Pontificia Universidad Catolica de Chile: performance of the Spanish translation of the Dundee Ready Education Environment Measure (DREEM). Educ Health.

[39] Özan S, Tımbıl S, Bilgin AC, Semin S (2015). The final step to becoming a physician: interns’ educational and working environment. Educ Sci.

[40] AAMC Medical School Graduation Questionnaire (2014). https://www.aamc.org/download/397432/data/2014gqallschoolssummaryreport.pdf.

[41] Boor K, Scheele F, van Der Vleuten CP, Teunissen PW, den Breejen EM, Scherpbier A (2008). How undergraduate clinical learning climates differ: a multi-method case study. Med Educ.

[42] Heck R (2000). Examining the impact of school quality on school outcomes and improvement: a value-added approach. Educ Adm Q.

[43] Goddard R, Hoy W, Hoy A (2000). Collective teacher efficacy: its meaning, measure, and impact on student achievement. Am Educ Res J.

[44] Ghaith G (2003). The relationship between forms of instruction, achievement and perceptions of classroom climate. Educ Res.

[45] Clifton RA, Perry RP, Stubbs CA, Roberts LW (2004). Faculty environments, psychosocial dispositions, and the academic achievement of college students. Res High Educ.

[46] Freiberg HJ (1998). Measuring school climate: let me count the ways. Educ Leadership.

[47] Till H (2004). Identifying the perceived weaknesses of a new curriculum by means of the Dundee Ready Education Environment Measure (DREEM) inventory. Med Teach.

[48] Al-Hazimi A, Al-Hayani A, Roof S (2004). Perceptions of the educational environment of the medical school in King Abdul Aziz University, Saudi Arabia. Med Teach.

[49] Bassaw B, Roff S, McAleer S, Roopnarinesingh S, Lisle J, Teelucksingh S (2003). Students’ perspectives on the educational environment, Faculty of Medical Sciences Trinidad. Med Teach.

[50] Demirören M, Palaoglu Ö, Kemahli S, Özyurda F, Ayhan HI (2008). Perceptions of students in different phases of medical education of educational environment: Ankara University Faculty of Medicine. Med Educ Online.

[51] Holt MC, Roff S (2004). Development and validation of the Anesthetic Theatre Educational Environment Measure (ATEEM). Med Teach.

[52] Henzi D, Davis E, Jasinevicius R, Hendricson W, Cintron L, Isaacs M (2005). Appraisal of the dental school learning environment: the students’ view. J Dent Educ.

[53] Ahmed AU (2005). Students’ perceptions of the learning climate. J Teach Assoc.

[54] Bandura AB, Bandura AB (1995). Exercise of personal and collective efficacy in changing societies. Self-efficacy in changing societies.

